# Constructing Robust Cooperative Networks using a Multi-Objective Evolutionary Algorithm

**DOI:** 10.1038/srep41600

**Published:** 2017-01-30

**Authors:** Shuai Wang, Jing Liu

**Affiliations:** 1 Key Laboratory of Intelligent Perception and Image Understanding of Ministry of Education, Xidian University Xi’an 710071, China.

## Abstract

The design and construction of network structures oriented towards different applications has attracted much attention recently. The existing studies indicated that structural heterogeneity plays different roles in promoting cooperation and robustness. Compared with rewiring a predefined network, it is more flexible and practical to construct new networks that satisfy the desired properties. Therefore, in this paper, we study a method for constructing robust cooperative networks where the only constraint is that the number of nodes and links is predefined. We model this network construction problem as a multi-objective optimization problem and propose a multi-objective evolutionary algorithm, named MOEA-Net_rc_, to generate the desired networks from arbitrary initializations. The performance of MOEA-Net_rc_ is validated on several synthetic and real-world networks. The results show that MOEA-Net_rc_ can construct balanced candidates and is insensitive to the initializations. MOEA-Net_rc_ can find the Pareto fronts for networks with different levels of cooperation and robustness. In addition, further investigation of the robustness of the constructed networks revealed the impact on other aspects of robustness during the construction process.

Existing studies have shown that complex networks are powerful models for depicting the interactions of natural and social systems^1^,^2^,^3^. The properties of complex networks have been discovered and studied include the scale-free property^4^, small-world property^5^, community property^6^, and so on. These properties have been found in many practical systems. For example, the topology of the Internet exhibits the scale-free property^7^,^8^ and social networks always have the small-world property^5^. Several related studies have shown the applicable value of complex network theories such as evolutionary dynamics^9^,^10^,^11^,^12^ and multi-scale flow systems^13^,^14^,^15^,^16^,^17^.

One important aspect of system performance is that it should maintain its functionality even when attacks or errors occur—namely, robustness—an aspect that has attracted much attention in recent decades^18^,^19^,^20^,^21^. The world urgently needs robust system designs that are resistant to unknown errors and attacks. Existing studies have focused intensively on enhancing the robustness of specific networks. Several techniques have been applied to improve the robustness of networks through topological rewiring under structural constraints (such as the degree distribution), including the greedy algorithm^19^, simulated annealing^20^ and evolutionary algorithms^21^. These algorithms have been verified as effective in obtaining highly optimized networks that are more resistant to attacks and errors.

In the meantime, to model the cooperative phenomena in human society, the emergence of cooperation^22^,^23^,^24^ has also been emphasized in recent researches. To express altruism and selfishness in economics and sociology, several evolutionary games such as the prisoner’s dilemma^22^ and the snowdrift game^23^ have been proposed to model the evolution of cooperation. In evolutionary game theory cooperators tend to obtain smaller profits while defectors obtain larger profits. Moreover, those with smaller profits are likely to be eliminated in the process of evolution. Previous studies have combined evolutionary games together with complex network theory^9^,^10^,^11^,^12^,^25^ to provide flexibility in realistic simulations and found that networks with different structures have different capabilities for preserving cooperators. This fluctuation in cooperation during an evolutionary process under topological changes has also attracted great attention in recent studies^26^,^27^,^28^,^29^. Due to its similarity with robustness evaluating network integrity, the robustness of cooperation suffers severely when hubs are lost, which provides an explanation for some types of social events. These studies have confirmed that network structure is key for maintaining cooperation and have revealed the internal relationship between robustness and cooperation in sociology and economics.

Generally, robustness design focuses on the integrity of entire networks, a property that is crucial for maintaining the functionality of systems under attacks or errors. On the other hand, cooperation emphasizes the behaviour of individuals and exists broadly in social dilemmas and economic activities. A robust structure is certainly necessary for decision makers; furthermore, considering the behaviours of members, maintaining a balance between cooperators and defectors is also important for system stability and harmony. Several realistic examples have revealed the significance of constructing robust cooperative systems.

The China West-East Electricity Transfer Project was designed to bring investment and development to China’s lagging western regions while satisfying the growing electricity needs of the country’s eastern provinces^30^,^31^. This effort requires the western Chinese provinces, where the population is low, to take advantage of their resource to generate massive amounts of electricity for the eastern provinces where the population is high. The project did improve the robustness of China’s electricity network, effectively solving the shortage of electricity in the densely populated areas in China and enhancing the fault tolerance of the power network. However, as indicated by some studies and news sources^32^,^33^,^34^, taking local economic profits into account, several eastern provinces in China have tended to refuse to purchase the electricity generated by the western provinces and, instead, have developed local electricity generation facilities. Unfortunately, for the western provinces, which have consumed large amounts of natural resources and suffered the effects of environmental pollution, this situation means that they cannot achieve sufficient economic benefit, while the eastern provinces have gained considerable economic profit from developing their local power industry. This outcome means the whole project is stuck in a dilemma that damages the balance of inter-regional development. In this case, if policy makers were provided with a set of power network structures with different performances of robustness and cooperation that have the potential to help maintain a balance between local profits and regional cooperation, planning power supplies between the different districts would be both more reasonable and more convenient.

Similarly, in biological relationship networks, biotic populations are often faced with the dilemma of balancing cooperation and competition^35^,^36^. If individuals in the population cooperate with one another, sharing their food, water, territory, and so on, the competitive pressure on the population tends to decrease, which, means that the excellence of the population cannot be maintained, eventually, the entire population tends to become less resistant to natural enemies or environmental disasters. On the other hand, if the population stresses competition too highly, none of the individuals cooperate with each other, which, although it generally elevates the fitness of the population, harms reproductive fitness because many individuals will fail to reproduce because of the fierce competition. Cooperation in biotic populations is common in nature, but when populations are rare and under-protected, animal wardens must intervene to raise the level of individual cooperation when necessary. Under these circumstances, a more rational distribution of individuals is needed to restore the balance between cooperation and the population robustness.

Additionally, in economics, different companies can choose to cooperate with each other or to compete. Too much cooperation causes a lack of innovation and makes industries less competitive against overseas competition and less resistant to financial turbulence, while too little cooperation leads to cutthroat competition, which is also not conducive to healthy development^36^,^37^. For industry regulators, there is an urgent need to negotiate a balance between a fully cooperative situation and cutthroat competition; in this regard, alternative networked structures of industrial members can be of help. Meanwhile, companies can adjust their strategies and benefit from more moderate structures that balance their performance between robustness and cooperation.

These examples reveal that evolutionary games exist broadly in daily life, and that policy makers are often faced with the task of adjusting the self-interested relationships among members while also improving the failure tolerance of the entire system. From the perspective of topological reshuffling, we try to construct networked systems with different levels of cooperation and robustness that can provide potential solutions to economic and social dilemmas. In the following experiments on real networks, we provide possible solutions to these examples through our proposed algorithm.

Existing studies on network-based cooperation have focused on modelling different types of evolutionary games and on researching the relations between topological features and the ability to maintain cooperation^9^,^10^,^11^,^12^,^22^,^23^,^24^,^25^; however, few have investigated techniques to promote the level of cooperation in networked systems, despite the fact that such techniques have potentially broad applications to real life. Based on previous studies, we focus on constructing networks that have a desired level of cooperation. Meanwhile, because both robustness and cooperation in complex networks are of great significance in real world applications and theoretical analyses, robustness has also been considered in the construction process. However, existing studies have shown that robustness and cooperation are in conflict with each other in terms of network structure. For robustness, an assortative or low-heterogeneity structure improves attack and error tolerances^18^,^19^,^38^, but such structures also constrain the emergence of cooperators^10^,^11^. To solve these problems, multi-objective optimization methods should be employed in the process of constructing robust cooperative networks. By doing so, we expect to generate a series of networks with different levels of robustness and cooperation.

In terms of topological reshuffling, structural rewiring methods that start from a given network have been shown to be effective in improving robustness^18^,^19^,^20^,^21^. Structural rewiring methods limit the degree distribution of optimized networks and must start from a specific initial network. However, these rewiring methods are helpless if the detailed connections in networks are unreachable; a method that can construct networks with the desired properties from arbitrary inputs is more flexible. Furthermore, considering the way cooperation emerges in the real world, it is not difficult to build connections with people we have never known before or to break off connections we already have, freeing from the total contacts we currently have (i.e., degree), as indicated by several existing studies^9^,^10^,^36^. Meanwhile, because the resources in a system are limited, the numbers of total cooperative relations for each individual and for the whole system are also restricted^36^,^37^ (i.e., it is impossible to generate cooperative connections between every pair of members). Therefore, in the process of constructing networks with a desired robust and cooperative level, we first define the number of nodes, representing that the system is relatively independent that no new members will join. Then, considering that allowing a variable number of links will make it difficult to evaluate and compare robustness as well as potentially creating confusing network structures, for simplicity, we also restrict the number of links during the construction process.

To sum up, in this paper, we model the issue of constructing robust cooperative networks as a multi-objective problem (MOP) and study the correlation between robustness and cooperation. Because evolutionary algorithms (EAs), which are optimization methods motivated by biological inheritance and evolution, have been shown to be highly efficient in solving MOPs^39^,^40^,^41^,^42^,^43^, we propose a multi-objective optimization algorithm based on the NSGA-II^40^ framework named MOEA-Net_rc_ to solve the modelled MOP. The performance of MOEA-Net_rc_ is validated on several widely-used network models, including Erdős-Rényi (ER) networks^44^, scale-free (SF) networks and small-world (SW) networks as well as several real-world networks. The results show that MOEA-Net_rc_ can construct networks with a balanced performance between robustness and cooperation from different initial network states. Furthermore, we conducted some comparative experiments to study the properties of the constructed networks on the obtained Pareto fronts.

## Results

A network can be represented as a graph *G* = (*V, E*), where *V* = {1, 2, …, *N*} is the set of *N* nodes and *E* = {*e*_*ij*_ | *i, j* ∈ *V*} is the set of *M* edges. In this paper, we focus on studying the robustness and cooperation of undirected networks.

### Robustness measures and optimization

A robust network tends to maintain functionality when failures occur on nodes or links. Several measures have been proposed recently to evaluate the robustness of networks from different aspects. One type of measures is based on the eigenvalue of Laplacian matrix^45^. Later, from the viewpoint of graph theory, Albert *et al*.^46^ proposed the robustness measure in which network disintegration was used to evaluate the performance of networks under critical conditions such as removal of some portions of the vertices or edges. In 2011, Schneider *et al*. proposed a novel measure, called *R,* which considers the largest connected component during all possible malicious attacks on nodes^19^ and is defined as follows:


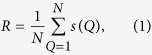


where *N* stands for the number of nodes in a network, *Q* stands for the number of nodes removed, and *s(Q)* stands for the fraction of the largest connected component after removing *Q* nodes. The normalization factor 1/*N* ensures that networks with different numbers of nodes are comparable. Because *R* exhibits good performance, we employ *R* to evaluate network robustness in this paper.

In addition to designing proper measures to evaluate network robustness, investigating how to improve network robustness is also important. Some optimization algorithms have been proposed to enhance the robustness of complex networks. Buesser *et al*. proposed a heuristic method based on simulated annealing to rewire the connections among nodes to obtain robust networks^20^. Except for local search methods, which are time consuming and have difficulty finding global optima, EAs have been employed to improve network robustness. For example, Zhou *et al*. proposed a memetic algorithm to optimize the robustness of networks^21^.

Additionally, structural measures also impact the robustness of networks. Herrmann *et al*. showed that networks with “onion-like” structures tend to perform better in resisting malicious attacks^18^. Assortativity (*r*)^47^,^48^ can evaluate the mixing patterns between connected nodes; networks with higher *r* tend to present “onion-like” structures and to possess high resistance to sustain attacks. Ma *et al*. indicated that the degree distribution of networks also affects their robustness; networks with nodes similar in degree show significantly higher tolerance to malicious nodal attacks^38^. In terms of degree distribution, the heterogeneity index (*H*)^49^ describes the level of inequality among degrees in networks; networks with smaller *H* tend to have a homogeneous degree distribution and show higher robustness.

### Evolutionary games on complex networks

The evolution of cooperation is a metaphor for social and biological activities. Several evolutionary games^22^,^23^ have been proposed to model the mutual relations involved in cooperation. The goal of these games is to reflect the strategies made by players through various resulting payoffs. In this paper, we focus mainly on the prisoner’s dilemma (PD) game^22^. The PD game clearly shows the payoff interaction between cooperators and defectors. Players choose their strategies (cooperate or defect) synchronously, and the payoff for each player is based on the choices of all that player’s neighbours. Each pair of players receives *P* for mutual defection and *R* for mutual cooperation. When the connected players make different choices, the defector obtains *T* and the cooperator obtains *S*. The order of payoff value is *T* > *R* > *P* > *S*. After all players achieve a payoff in a system, an update operation is conducted. Players with higher payoffs tend to influence the strategies used by their neighbours. In networking terms, as described in refs 11 and 27, for every node *i* in the network, randomly choose a neighbour *j*, and denote the payoff of node *i* as *P*_*i*_. If *P*_*i*_ < *P*_*j*_, node *i* imitates the strategy of node *j* with the probability 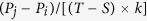, where *k* is the largest degree between nodes *i* and *j*. The update operation should be performed numerous times until the system reaches a stable state. Then, in this paper, the fraction of cooperators remaining in the network, labelled *f*(*c*), is used as the measure that evaluates a network’s ability to maintain cooperation.

Previous studies have illustrated that disassortative and heterogeneous structures show a high ability to maintain cooperation^10^,^11^ because fewer connections between hubs and the existence of cooperative hubs reduce the possibility of cooperators being invaded. On the other hand, it is also feasible to regulate network structures under the guidance of some structural measures to reach different levels of cooperation, which is the approach adopted in this paper.

### Correlation between robustness and cooperation

As discussed previously, a network’s structure is critical to both its robustness and its level of cooperation. To construct desirable networks, we can model the construction task as an optimization problem. In optimization problems, the objectives by which selections are made are important. Focusing on constructing robust cooperative networks, we can take robustness and cooperation as two objectives, and model the task as an MOP^50^. Thus, we first analyse the correlation between these two objectives through the following experiment.

In MOPs, contradictions should exist in the optimization objectives; otherwise, the problem could be solved as a single optimization problem. In this experiment, we take SF networks as an example to show the correlation between the robustness and cooperation. Because calculating the *f*(*c*) of a network is time consuming, we use *R* as the tuning target—that is, we generate networks with both high and low *R*, and then, the *R* and *f*(*c*) of each network are calculated to study their correlation. Similar to the work of Ma *et al*. in ref. 38, the *heuristic optimization method* is implemented to rewire the connections between nodes to adjust the objective *R* by changing the degree distribution of networks. When evaluating *f*(*c*), we set the payoff parameters as *T* = 2, *R* = 1, *P* = 0, and *S* = 0. The details of this method are as follows.

**Figure 1:**
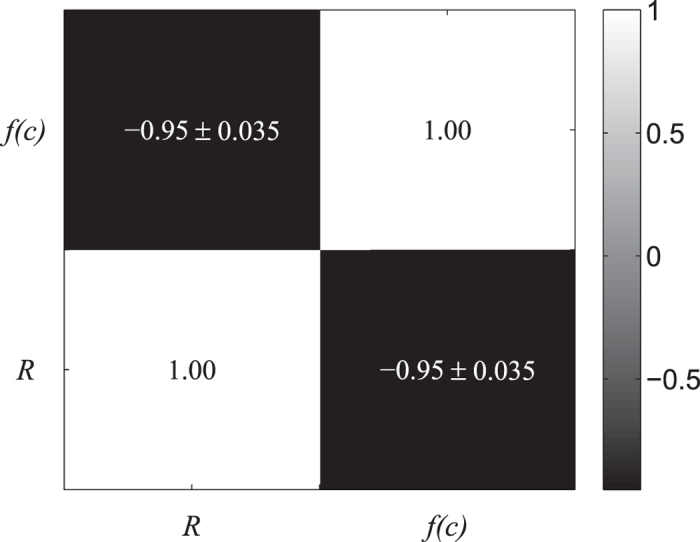
The Pearson correlation coefficient between *R* and *f*(*c*).

1. Based on the BA model^4^, generate an SF network *G* randomly;
2. Take *R* as the objective function, and use the *heuristic optimization method* to generate networks with minimum *R*. When the *R* of the current network does not decrease over 1000 evaluations, assign the current network to *G*_*ori*_;
3. Optimize the *R* of *G*_*ori*_ using the *heuristic optimization method*; when the *R* of the current network increases by 5%, sample this network and continue the optimization process until *R* remains unchanged for 1000 evaluations;
4. Conduct the evolutionary game on the obtained networks to calculate the *f*(*c*) of each network;
5. Repeat the above steps ten times; the average results are shown in Fig. 1.

As shown in Fig. 1, the Pearson correlation coefficient between *R* and *f*(*c*) shows that these two objectives are strongly negatively correlated with each other on SF networks, which indicates that the multi-objective optimization model is meaningful in modelling the task of constructing robust cooperative networks.

Furthermore, *R* and *f*(*c*) also show different correlations in different network structures. Here, we use heterogeneity (*H*) and assortativity (*r*) to evaluate the structural property of networks. As Hu *et al*. proposed in ref. 49, the heterogeneity (*H*) of networks is defined as follows:


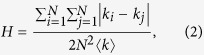


where *N* is the number of nodes in a network, *k*_*i*_ represents the degree of node *i*, and 〈*k*〉 is the average degree of the network. *H* lies in the range of [0, 1], and larger values imply higher heterogeneity in terms of network degree. Newman proposed the assortativity property (*r*) in ref. 47, which evaluates the propensity with which vertices of similar degree are connected to one another. Assortativity is defined as follows:


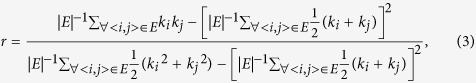


where |*E*| is the total number of links in a network, *k*_*i*_ is the degree of vertex *i*, and the value of *r* is equal to the Pearson correlation coefficient of the degrees.

We adjust *H* and *r* to diversify the structures of specific SF networks with *N* = 200, a scaling exponent^51^,^52^ of *α* = 3 and 〈*k*〉 = 4. In the PD game, we set *T* = 2, *R* = 1, and *P* = *S* = 0. The correlations between these measures and the two objectives can be seen in Supplementary Figs. S1 and S2 where mutual promotion exists between *H* and *f*(*c*), but a contradictory relationship exists between *H* and *R*, which indicate that the degree distribution of networks impact robustness and cooperation separately. Similarly, an increase in *r* limits the existence of cooperators but promotes the ability of network to resist malicious attacks and vice versa.

From the above experiments, we can see that constructing networks with better robustness and high ability in maintaining cooperation are conflicting objectives. Thus, we model this task as an MOP, and take *R* and *f*(*c*) as the two objectives.

### Experimental results on synthetic networks

In this section, the performance of MOEA-Net_rc_ is validated on synthetic networks. To validate the effect of different initial networks on the performance, three types of networks are taken as initial networks: ER networks generated by the Erdős-Rényi model^44^, SF networks generated by the BA model^4^, and SW networks generated by the WS model^5^. Then, the Pareto fronts obtained by MOEA-Net_rc_ are analysed. Moreover, the topology features of networks on the Pareto fronts such as the largest number of connected components and other robustness measures are also studied with the goal of investigating the changes in functionality from diverse aspects.

**Figure 2:**
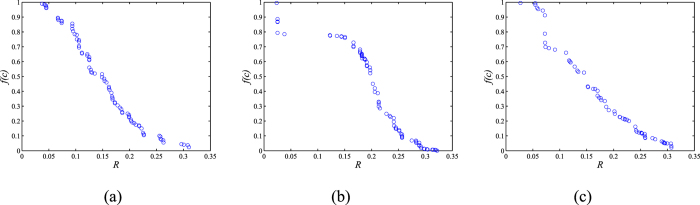
The Pareto fronts obtained by MOEA-Net_rc_ from different initial synthetic networks with 〈*k*〉 = 4. (**a**) ER-initial, (**b**) SF-initial, and (**c**) SW-initial.

Synthetic networks with size *N* = 200 are used in this experiment. The effect of degree-density on network performance is also studied by using networks with different average degrees, 〈*k*〉. In the experiment, we set *T* = 2, *R* = 1, *P* = 0, and *S* = 0 when conducting the evolutionary game. The obtained Pareto fronts from different initial networks with 〈*k*〉 = 4 are shown in Fig. 2 and the results of networks with 〈*k*〉 = 8 are depicted in Supplementary Fig. S3.

As shown in Fig. 2 and Supplementary Fig. S3, the obtained Pareto fronts provide networks with balanced *R* and *f*(*c*) properties. Decision makers can make choices according to different practical requirements. Focusing on the Pareto fronts obtained from the three types of initial networks, their continuities have slight differences—mainly due to the structural diversity of the initial networks. In general, MOEA-Net_rc_ can construct desired networks with different levels of *R* and *f*(*c*) from arbitrary initializations under constraints where the numbers of nodes and links are predefined. Previous studies^4^,^5^,^44^ have indicated that most real networks can be modelled as ER, SF, or SW networks. MOEA-Net_rc_ is effective on all these models when dealing with the problem of constructing robust cooperative networks that possesses the potential to be used in practical applications. In addition, for testing the performance of MOEA-Net_rc_ on different sizes of networks, the obtained Pareto fronts from SF-initial (〈*k*〉 = 4) networks with large sizes are shown in Supplementary Fig. S4. The results show that MOEA-Net_rc_ is still effective for larger-sized networks.

Next, we take SF-initial networks as an example to analyse the ranges of *H* and *r* of the networks on the Pareto fronts. The results are shown in Supplementary Fig. S5. In the initial stage, we adopt heterogeneity (*H*) to make the network structures diverse; then, in the later stage, assortativity (*r*) has also been considered to further adjust the structure of specific networks. As shown in Supplementary Fig. S5(a) and (b), networks with heterogeneous structures (larger *H* values) tend to promote the emergence of cooperation, while networks with homogeneous structures (smaller *H* values) tend to enhance network robustness. Through this construction process, a series of networks possessing different *H* values are generated. Moreover, the population is initialized with candidates that fall into different levels of the two objectives, which accelerates the convergence of the searching process. In addition, the results in Supplementary Fig. S5(c) and (d) validate the relation between *r* and the two optimization objectives. Assortative networks with higher values of *r* tend to show resistance against malicious attacks, but disassortative networks with lower values of *r* support the existence of cooperators in evolutionary games. On the other hand, evaluating *f*(*c*) is time consuming, but adjusting *r* provides potential individuals with the desired ability to maintain cooperation and also contributes toward improving the convergence speed of the algorithm. These results depict the relationship between network properties and the optimization objectives on the networks located at Pareto fronts, which conforms to the conclusions in the previous analyses and reveals the properties of networks with different levels of *R* and *f*(*c*).

Because adjusting *H* and *r* may also obtain networks with desired levels of robustness and cooperation, we compared the networks obtained by adjusting only *H* and *r* with those on the obtained Pareto fronts. We selected networks with a desired level of heterogeneity and adjusted assortativity to obtain the desired networks. The results in Supplementary Fig. S6 show that the networks generated by adjusting only *H* and *r* are mainly distributed randomly in the searching space, and they fluctuate within a relatively small range; however, they are much worse than the networks on the Pareto fronts found by our algorithm. Thus, simply adjusting *H* and *r* cannot generate networks with the desired properties, and the multi-objective model is a good solution for this problem.

In terms of PD games, we tested the situation with *T* = 2, *R* = 1, *P* = 0, and *S* = 0, which is weak PD game^10^,^23^. For comparison, we also conducted the experiment with *T* = 2, *R* = 1.5, *P* = 0, and *S* = 0 (here, 2 *R* > *T*) on SF-initial networks with *N* = 200. The results are shown in Supplementary Fig. S7. When compared with the results in Fig. 2(b) and Supplementary Fig. S3(b), the Pareto fronts are almost the same, which indicates that the cases of 2 *R* > *T* and 2 *R* = *T* in the PD game show similar performance in the construction process.

Because the Pareto Fronts in Fig. 2 provide a set of networks with different performance in terms of the two objectives, we extracted networks from different parts of Pareto fronts and analysed their topologies. Following Zhou *et al*.^53^, three networks are extracted from each Pareto front (for visual effect, we took networks with 〈*k*〉 = 4 as examples here). The network with the largest *R* is labelled *G*_*r*_ and is located in the right part of the Pareto front; the network with the largest *f*(*c*) is labelled *G*_*l*_ and is located in the left part of the Pareto front; and the balanced network is labelled *G*_*m*_ and is located in the middle of the Pareto front. The topologies of these three networks are shown in Supplementary Fig. S8. The networks structural results show that, for these three types of initial networks, the algorithm achieves the purpose of adjusting structures to construct the desired networks and the degree distributions of *G*_*l*_, *G*_*m*_ and *G*_*r*_ are significantly different. In addition, the number of links between nodes with similar degree has been increased in the process of constructing robust networks. Furthermore, we analysed the numeric properties of *G*_*l*_, *G*_*m*_ and *G*_*r*_. As listed in Table 1, the results show that networks with higher levels of cooperation tend to possess a larger *H* and a smaller *r* and always have star-like structures. In contrast, those with higher robustness tend to possess a smaller *H* and a larger *r* and mostly have an onion-like structure (similar to the conclusions in refs 18 and 19). Networks with trade-offs between the two extreme structures show balanced performances in terms of *R* and *f*(*c*).

**Figure 3:**
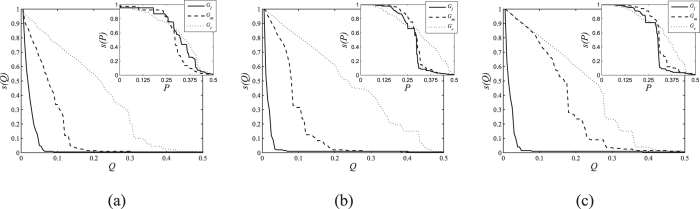
The fraction of the largest connected sub-graphs obtained during nodal and edge-based attacks. (**a**) ER-initial networks, (**b**) SF-initial networks, and (**c**) SW-initial networks.

To further investigate the different performances of these networks, we studied the largest connected components during the process of attacks on the selected networks shown in Fig. 3, taking both the node-degree and the link-degree as indicators. The fraction of the largest connected sub-graphs obtained after nodal attacks and edge-based attacks are labelled as *s*(*Q*) and *s*(*P*), respectively. The numeric results are shown in Fig. 3.

As shown in Fig. 3, during the node-removal process, different initial states of networks show similar performances. The results in Figs 2 and 3 and in Supplementary Fig. S3 illustrate that MOEA-Net_rc_ has good generalization ability over different initial networks, and can construct candidate networks with different values of *R* and *f*(*c*). However, the results in Fig. 3 also indicate that the constructed networks show few differences with regard to the results of link attacks.

**Figure 4:**
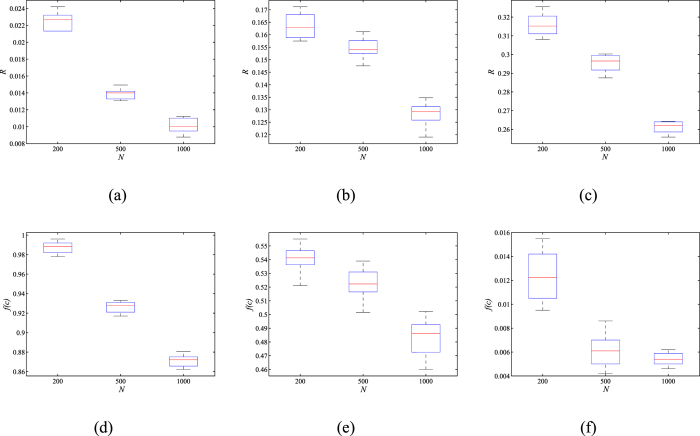
The mean and variance of *R* and *f*(*c*) of the selected networks on the Pareto fronts. The images in (**a**), (**b**), and (**c**) represent the numeric results of *R* on *G*_*m*_, *G*_*l*_, *G*_*r*_, respectively, while (**d**), (**e**), and (**f**) represent the results of *f*(*c*) on *G*_*m*_, *G*_*l*_, *G*_*r*_. The results are averaged over 20 independent realizations.

To test the stability of MOEA-Net_rc_, we took the SF-initial networks as an example. The number of nodes is set up to 1000, and 〈*k*〉 = 4. *G*_*l*_, *G*_*m*_ and *G*_*r*_ are extracted from each Pareto front. The mean and the variance of *R* and *f*(*c*) shown in Fig. 4 indicate that the performance of MOEA-Net_rc_ is stable.

In the above experiments, we focused on solving the MOP over *R* and *f*(*c*) with MOEA-Net_rc_, obtaining a set of networks with different values of *R* and *f*(*c*) on the Pareto fronts. However, there are many measures other than *R* to evaluate the robustness of networks. The networks on the SF-initial Pareto fronts in Fig. 2 are evaluated by some of these other robustness measures, and the corresponding numeric results are reported in Supplementary Fig. S9. As seen from the results, the optimized networks on the Pareto fronts have correlations with other robustness measures that reveal the internal relations between *R* and these measures.

### Experimental results on real-world networks

In this section, we validate the performance of MOEA-Net_rc_ on three real-world networks, namely, the WU-Power grid network, Dolphin social network, and Scotland corporate interlock network. (see Supplementary Table S1 for detailed information).

**Figure 5:**
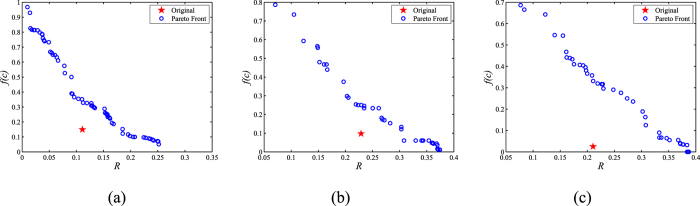
The Pareto fronts obtained by MOEA-Net_rc_ on real-world networks. The corresponding *R* and *f*(*c*) values of the original networks are represented as red stars. (**a**) shows the result for the WU-Power grid network, (**b**) shows the Dolphin social network result, and (**c**) shows the result for the Scotland corporate interlock network.

The Pareto fronts obtained by MOEA-Net_rc_ are depicted in Fig. 5, and the *R* and *f*(*c*) values of the original networks are depicted as red stars. The results show that the obtained Pareto fronts have good distributions and generate a set of networks with different performances in terms of *R* and *f*(*c*). Compared with the results in ref. 53, MOEA-Net_rc_ can achieve a wider scope for *R*, which can provide more useful choices for decision makers in real applications.

Similar to the analyses on synthetic networks, *G*_*l*_, *G*_*m*_ and *G*_*r*_ are extracted from the Pareto fronts in Fig. 5 to show the structures of optimized networks, the results are given in Supplementary Fig. S10. In the experiments on real networks, we achieve similar structural fluctuations as those in Supplementary Fig. S8. Decision makers can select suitable networks from these results based on their needs. For power networks, a structure that exhibits both robustness and maintains the potential cooperation may be helpful for solving the dilemma between local interests and the overall stability of the power supply system discussed in refs 32, 33, 34. For biological relations networks, animal wardens can intervene into the relationships between individuals based on the aforementioned results to balance the emergence of cooperation and competition of the entire biotic population to facilitate the reproduction of protected or rare species. For corporate interlock networks, industry regulators will benefit from the construction results by acquiring solutions that address malicious developing situations, meanwhile company owners can also adjust their operational strategies by referring to the results to avoid excessive competition and develop wiser tactics.

In this section, we tested the performance of MOEA-Net_rc_ on three real-world networks, and the results show that MOEA-Net_rc_ can find valuable potential solutions to the multi-objective problem of constructing robust cooperative networks, which may be of help in solving real-world dilemmas in sociology and economics.

## Discussion

The emergence of cooperation and the ability to withstand attacks are of great significance to networked systems: both properties should be taken into consideration in practical applications. Previous studies have indicated that networks with dense and homogeneous structure tend to have better robustness, but those with spare and heterogeneous structure help guarantee the emergence of cooperators in evolutionary games. Based on these conclusions, we illustrate the dilemmas between robustness and cooperation in real world through several realistic examples and model the problem of constructing robust cooperative networks as an MOP in this paper. Then, we devise a multi-objective EA, named MOEA-Net_rc_, to find a series of candidates for theoretical and potentially practical applications.

The contributions of this paper are summarized as follows: (1) Previous studies have proposed evolutionary game models that revealed the relation between network structural features and the ability to maintain cooperation, but designing networks with a specific cooperative level is still an open question. Based on these studies, we have achieved a method for building networks with a desired level of cooperation using topological rewiring. The constructed networks can facilitate both theoretical analyses and potential applications. (2) Constructing networks with high attack tolerances—a property crucial to networked systems—has also been considered in this paper. Because several recent examples have revealed a trade-off between the properties of robustness and cooperation is of significance, we model the problem for constructing networks with both high robustness and a good ability to maintain cooperation maintaining as an MOP. Then, we propose MOEA-Net_rc_ to solve the MOP. In addition, considering the large computational cost required to evaluate the level of cooperation in a network, we designed an effective initialization operator for MOEA-Net_rc_ that can obtain a broad distribution of individuals in the solution space. This approach both provides potential candidates and accelerates the convergence of searching process. (3) Experimental results on synthetic networks—including networks generated from ER, SF and SW models—show that MOEA-Net_rc_ has good generalization ability. Finally, we tested the performance of MOEA-Net_rc_ on several real-world networks and achieved good solutions that can provide decision makers with candidates to help solve real-world dilemmas.

In addition to the single networks studied in this paper, the robustness and the emergence of cooperation in interdependent and coupled networks has attracted increasing attention in recent studies^54^,^55^,^56^. Therefore, our future work will involve further studying the construction of robust cooperative interdependent or coupled networks.

## Methods

We depicted the conflict between *R* and *f*(*c*) and modelled the simultaneous enhancing robustness and cooperation problem as an MOP. Because EAs have been widely employed in dealing with MOPs in the past few decades^40^,^41^,^42^,^43^, we propose a multi-objective evolutionary algorithm, named MOEA-Net_rc_, to solve the modelled MOP.

Usually, the initial population for an EA is generated randomly in the search space; however, in network construction problems, the search space is very large. If we were to generate the initial population randomly, the performance of most individuals in each objective would be very poor. Previous studies have shown that generating good initial populations by considering the optimization objectives rather than simply generating random populations can speed up the convergence of EAs and reduce the computational cost^53^,^57^. In ref. 53, Zhou *et al*. proposed a two-phase multi-objective EA to solve MOPs. On one hand, this approach generates initial populations whose individuals have widely distributed values in each objective. On the other hand, it addresses the problem that the computational costs of the two objectives are significantly different.

In our MOP, the computational costs for calculating *R* and *f*(*c*) are also significantly different because updating operations must be performed numerous times on networks to reach a stable state and determine *f*(*c*). Thus, we borrowed the concept from^53^ to first generate a well distributed initial population with different structural properties to reduce the computational cost and speed up the convergence. The heterogeneity of networks shows different correlations with the two objectives, so we took *H* as the property measure to adjust the structure of networks to obtain good initial populations for optimizing both *R* and *f*(*c*) and, thus, reducing the computational cost for determining *f*(*c*).

### Initialization stage

In MOEA-Net_rc_, graphs are presented as chromosomes. Thus, a population with *Ω* chromosomes represents *Ω* graphs labelled *G*_1_, *G*_2_,…, *G*_*Ω*_. We want to generate a widely distributed population by adjusting the *H* value from a specific network to modulate network structure. As shown in Supplementary Fig. S1, for a specific network, a heterogeneous structure with a larger *H* tends to promote *f*(*c*) but restrain *R* and vice versa. From this perspective, a population with a wide *H* distribution can provide balanced initializations for solving the MOP.

To modulate *H*, we employ the *heuristic optimization method* in ref. 38 by setting *H* as the optimization objective. The selections of pairs of edges are random, and the reconnection operation keeps the number of links unchanged. The details of the initialization stage are summarized in Algorithm 1. In the experiments, different initial states of networks have different ranges of *H*; the boundaries for *H* are determined by trial and error.


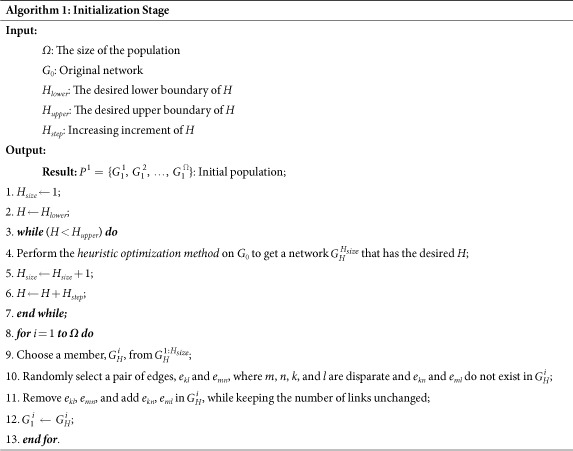


### Implementation of MOEA-Net_rc_

EAs have become popular approaches to solving MOPs^39^,^40^,^41^,^42^,^43^ in the past few years. Among existing EAs, NSGA-II^40^ showed an excellent performance; therefore, we implemented MOEA-Net_rc_ using the NSGA-II framework. In the basic process of NSGA-II, a *fast non-dominated sort* is employed to divide the population into different ranks. When optimizing *R* and *f*(*c*), it is desirable to obtain the maximum solutions in terms of each objective; consequently, if the values of both objectives of an individual *i* are smaller than those of individual *j*, then *i* is dominated by *j* and given a higher rank than *j*. Individuals with lower (better) ranks tend to be selected into the next generation. When both solutions have the same rank, the *crowd* should be compared, as described in ref. 40, using the *Crowded-Comparison Operator*, in which solutions that are located in less crowded regions are preferred in the selection.

In MOEA-Net_rc_, considering the broad distribution of the degrees of the initial populations, we avoid using the crossover operation between chromosomes, which is inefficient and fallible. The results in Supplementary Fig. S2 show that the assortativity (*r*) of networks presents correlates differently with *R* and *f*(*c*); thus, we designed different strategies for the two objectives. When searching for better solutions in terms of *R*, the *heuristic optimization method*^38^ is employed, which takes *R* as the optimization objective. In contrast, when optimizing *f*(*c*), the *rewire connection strategy* (following Buesser *et al*. in ref. 16) is employed, which rewires the network to adjust the *r* value of the current networks. In addition, to reduce the computational cost, an external population (*EP*) is used to store the non-dominated solutions found during the searching process (similar to Zhang *et al*. in ref. 41). In each generation of MOEA-Net_rc_, we update the *EP* using the current individuals. The details of MOEA-Net_rc_ are summarized in Algorithm 2.


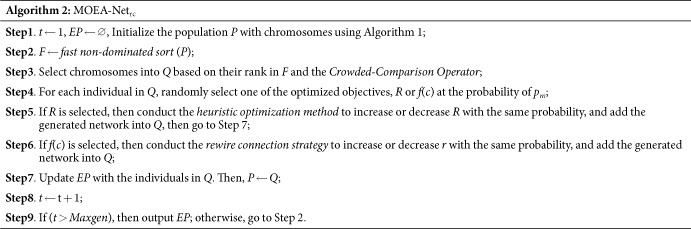


The parameters used in MOEA-Net_rc_ are described in Supplementary Table S2.

## Additional Information

**How to cite this article**: Wang, S. and Liu, J. Constructing Robust Cooperative Networks using a Multi-Objective Evolutionary Algorithm. *Sci. Rep.* **7**, 41600; doi: 10.1038/srep41600 (2017).

**Publisher's note:** Springer Nature remains neutral with regard to jurisdictional claims in published maps and institutional affiliations.

## Supplementary Material

Supplementary Materials

## Figures and Tables

**Table 1:** The numeric properties of *G*_*l*_, *G*_*m*_ and *G*_*r*_.

	Type	*H*	*r*	*R*	*f*(*c*)
*G*_*l*_	ER	0.5583 ± 0.011	−0.3324 ± 0.025	0.0371 ± 0.007	0.9925 ± 0.003
	SF	0.5061 ± 0.012	−0.4023 ± 0.033	0.0222 ± 0.003	0.9951 ± 0.002
	SW	0.5215 ± 0.009	−0.3932 ± 0.029	0.0265 ± 0.006	0.9950 ± 0.002
	Average	0.5286	−0.3760	0.0286	0.9942
*G*_*m*_	ER	0.4075 ± 0.025	−0.1676 ± 0.053	0.1527 ± 0.011	0.5312 ± 0.047
	SF	0.3825 ± 0.067	0.1251 ± 0.025	0.1638 ± 0.015	0.5211 ± 0.095
	SW	0.3671 ± 0.045	−0.1492 ± 0.046	0.1448 ± 0.016	0.5261 ± 0.076
	Average	0.3857	−0.0639	0.1538	0.5261
*G*_*r*_	ER	0.2155 ± 0.009	0.6533 ± 0.007	0.3122 ± 0.009	0.0562 ± 0.002
	SF	0.1713 ± 0.010	0.6131 ± 0.007	0.3159 ± 0.013	0.0142 ± 0.007
	SW	0.1573 ± 0.011	0.5190 ± 0.009	0. 3063 ± 0.011	0.0213 ± 0.004
	Average	0.1814	0.5951	0.3115	0.0306

The row labelled “Average” shows the mean values of all three types of networks.
