# Supplementary material for: Constructing Robust Cooperative Networks using a Multi-Objective Evolutionary Algorithm
Source: Sci Rep. 2017 Jan 30;7:41600. doi: 10.1038/srep41600 (PMC5278550; doi:10.1038/srep41600)
Supplement: Supplementary Materials [file srep41600-s1.pdf]

Supplementary Materials for Constructing Robust Cooperative Networks using a Multi-objective Evolutionary Algorithm

Shuai Wang[1] and Jing Liu[1]

Key Laboratory of Intelligent Perception and Image Understanding of Ministry of Education, Xidian University, Xi'an 710071, China. Correspondence and requests for materials should be addressed to Jing Liu (email: neouma@163.com)

Contents

1. **Supplementary Note 1: Other network robustness measures**

2. **Supplementary Figures**

3. **Supplementary Table**

4. **Supplementary References**

Supplementary Note 1: Other network robustness measures

Some other robustness measures are introduced in this section, and these measures are employed to evaluate the robustness of networks on the Pareto fronts for further depict the performance of the networks on Pareto fronts.

Zeng *et al.* extended R to the case of link-attacks in [S1] as follows,

$$R_l = \frac{1}{M}\sum_{P=1}^{M} s(P), \tag{S1}$$

where M is the number of links, $s(P)$ is the fraction of largest connected component after removing P links. $1/M$ is the normalization factor.

Besides, Wu *et al.* [S2] proposed the robustness measure based on the average eigenvalue of networks, termed as natural connectivity ($\bar{\lambda}$),

$$\bar{\lambda} = \ln\left(\frac{1}{N}\sum_{i=1}^{N} e^{\lambda_i}\right), \tag{S2}$$

where λ_i is the i-th eigenvalue of adjacency matrix.

Moreover, the communication efficiency between nodes in networks may also be influenced by the fluctuation of network structure, which is important in real-world communication and transportation networks. As Latora *et al.* proposed in [S3], the communication efficiency $C(G)$ considers the reciprocal of shortest path between nodes i and j in networks,

$$C(G) = \frac{1}{N(N-1)}\sum_{i\neq j} c_{ij} = \frac{1}{N(N-1)}\sum_{i\neq j}\frac{1}{d_{ij}}, \tag{S3}$$

where $c_{ij} = 1/d_{ij}$ and d_{ij} is the length of the shortest path between nodes i and j. When the two nodes belong to different components, $c_{ij} = 0$. Being similar with R, we take the sum of $C(G)$ during the process of attack as the measure:

$$ComE = \frac{1}{N}\sum_{q=1}^{N} C(G_q), \tag{S4}$$

where $C(G_q)$ is the communication efficiency of networks after removing q nodes, and $1/N$ is the normalization factor.

Supplementary Figures

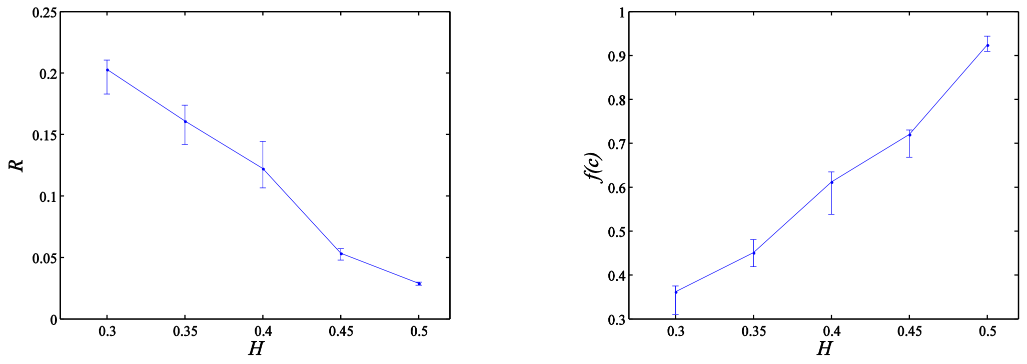

(a) (b)

Supplementary Figure S1. The correlation between (a) H and R, and (b) H and $f(c)$. The results are conducted on SF networks with 200 nodes and averaged over 20 independent realizations.

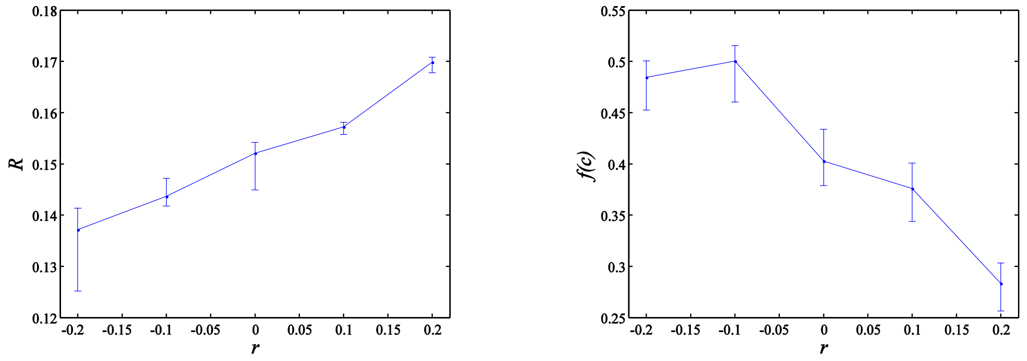

(a) (b)

Supplementary Figure S2. The correlation between (a) r and R, and (b) r and $f(c)$. The results are conducted on SF networks with 200 nodes and averaged over 20 independent realizations.

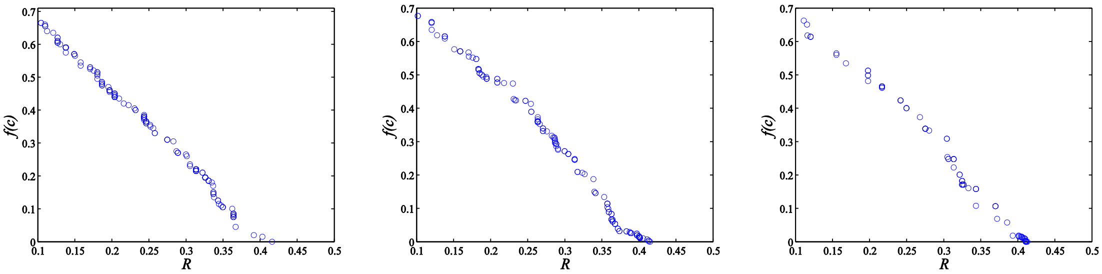

(a) (b) (c)

Supplementary Figure S3. The Pareto fronts obtained by MOEA-Net$_{rc}$ from different initial synthetic networks with $\langle k \rangle = 8$: (a) ER-initial, (b) SF-initial, and (c) SW-initial.

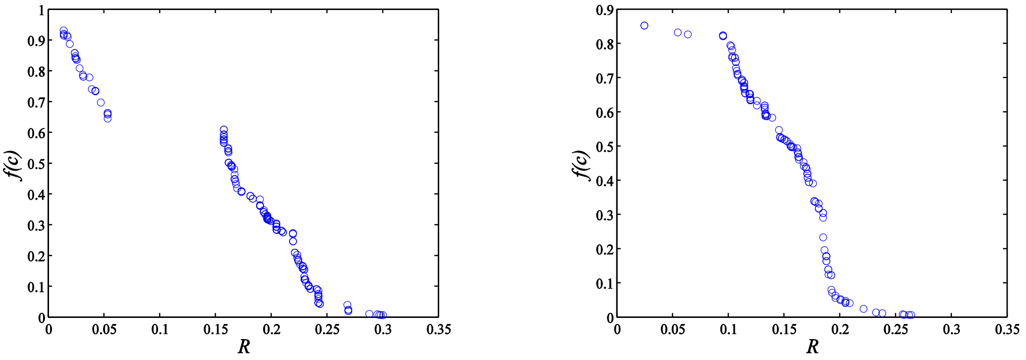

(a) (b)

Supplementary Figure S4. The Pareto fronts obtained by MOEA-Net$_{rc}$ from large size SF-initial networks: (a) N = 500, (b) N = 1000.

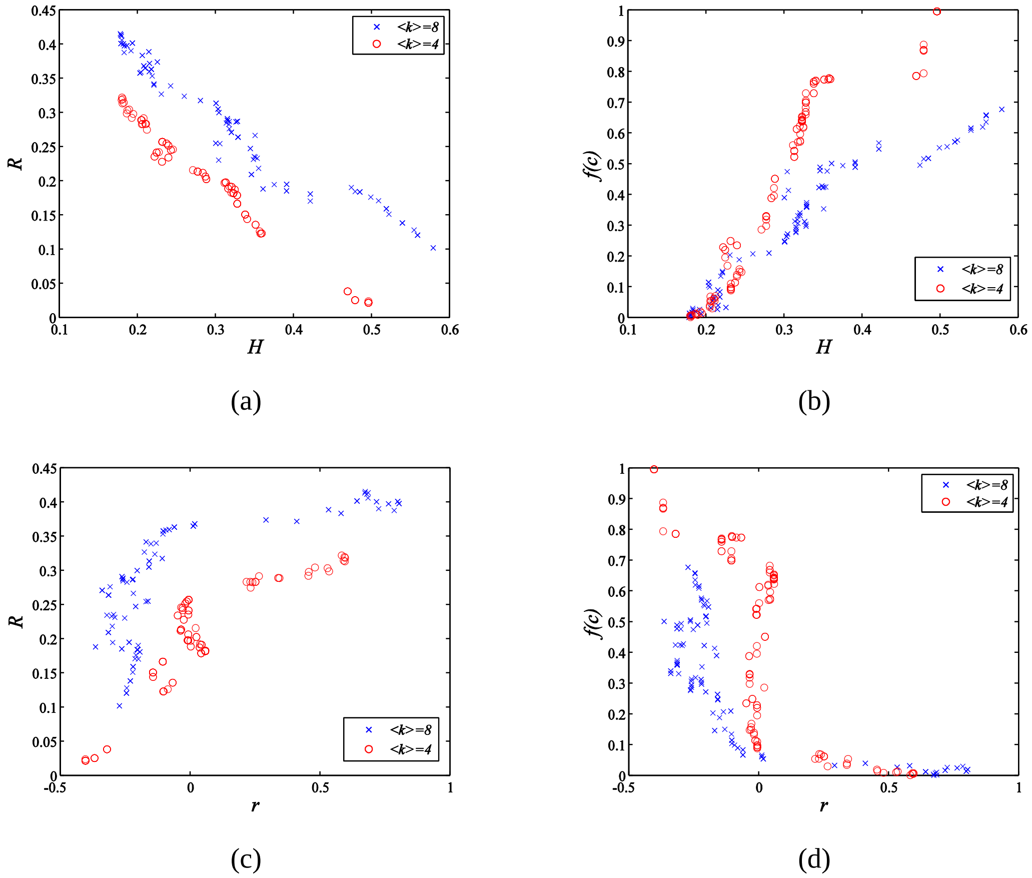

Supplementary Figure S5. The analyses of generated networks on the Pareto fronts of SF-initial network with N = 200 (a) shows results of H versus R, (b) shows results of H versus $f(c)$, (c) shows results of r versus R, (d) shows results of r versus $f(c)$.

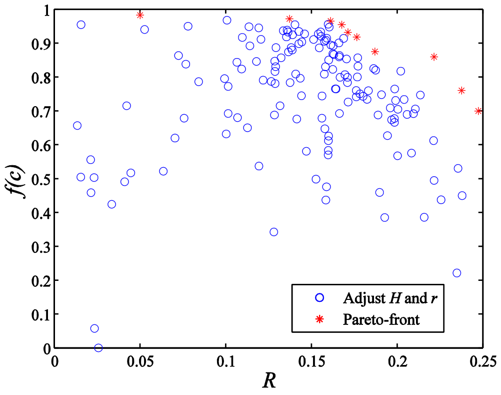

Supplementary Figure S6. The networks obtained by adjusting H and r and those on the Pareto fronts. The experiments are conducted on SF network with N = 200 and $\langle k \rangle$ = 4. The results of adjusting H and r just distribute randomly in the searching space and fluctuate in a relatively small range, which reveals the multi-objective model is a good solution for constructing robust cooperative networks.

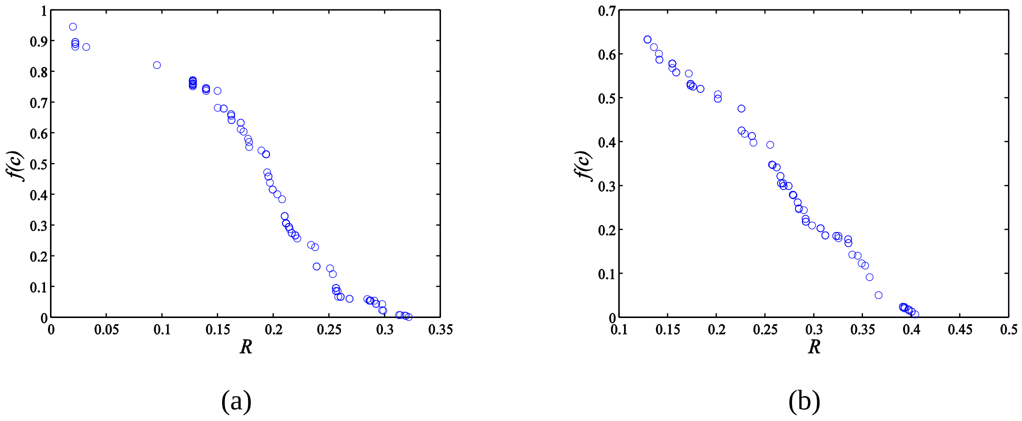

Supplementary Figure S7. The Pareto fronts of PD games under the case of $2R > T$ (the profit parameters in PD game are set as $T = 2$, $R = 1.5$, $P = 0$, and $S = 0$), the experiment is conducted on SF networks with $N = 200$ with (a) $\langle k \rangle = 4$ and (b) $\langle k \rangle = 8$.

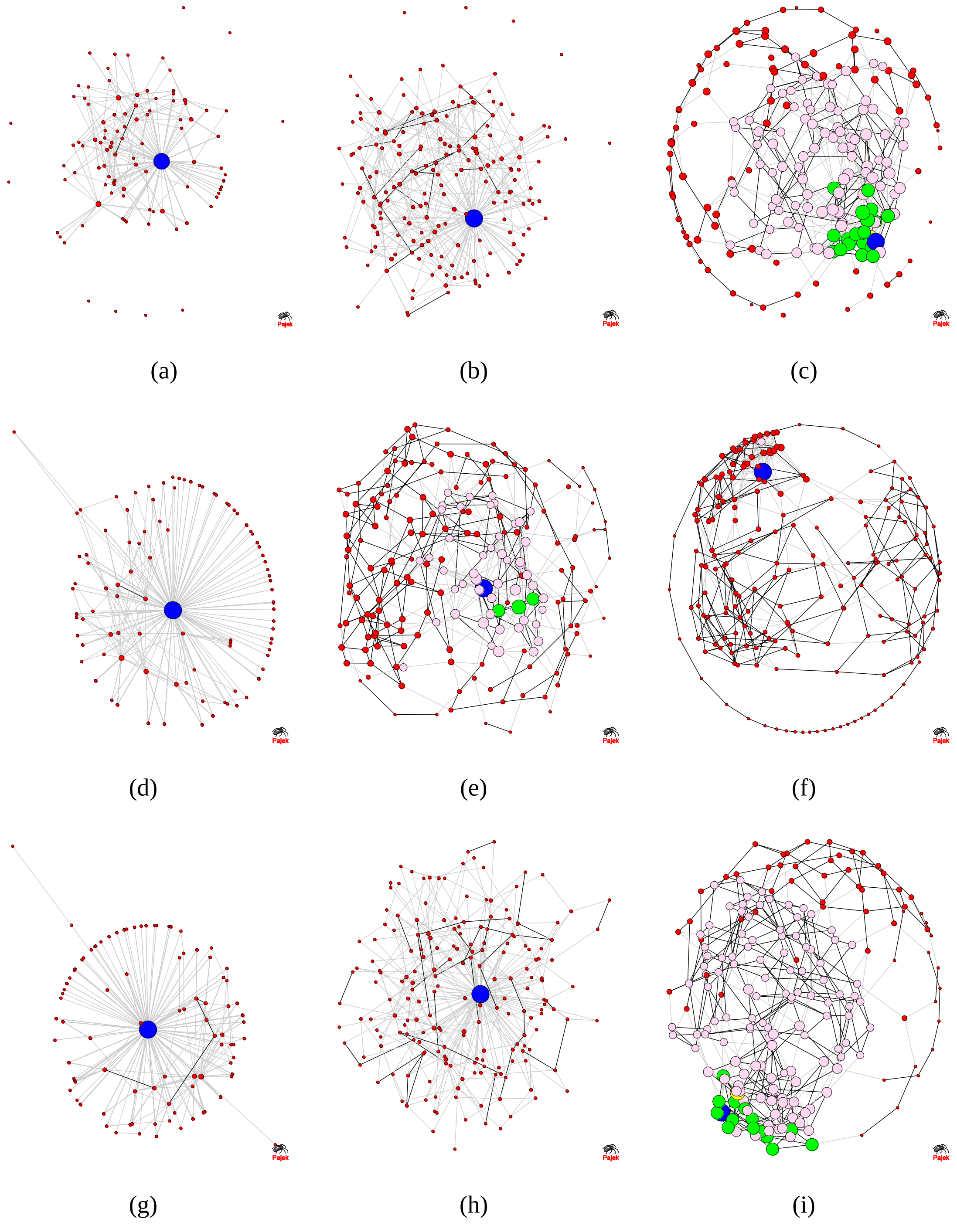

Supplementary Figure S8. Topologies of extracted networks from the Pareto fronts in Fig. 2. Images in the first row ((a), (b), and (c)) are extracted from Pareto front initialized by ER networks, in the second row ((d), (e), and (f)) are initialized by SF networks, and in the last row ((g), (h), and (i)) are initialized by SW networks. Images in the first column represent G_l, in the second column represent G_m, and in the last column represent G_r. In the figures, the size of nodes is proportional to the degree of this node. Links between nodes with same degree are highlighted.

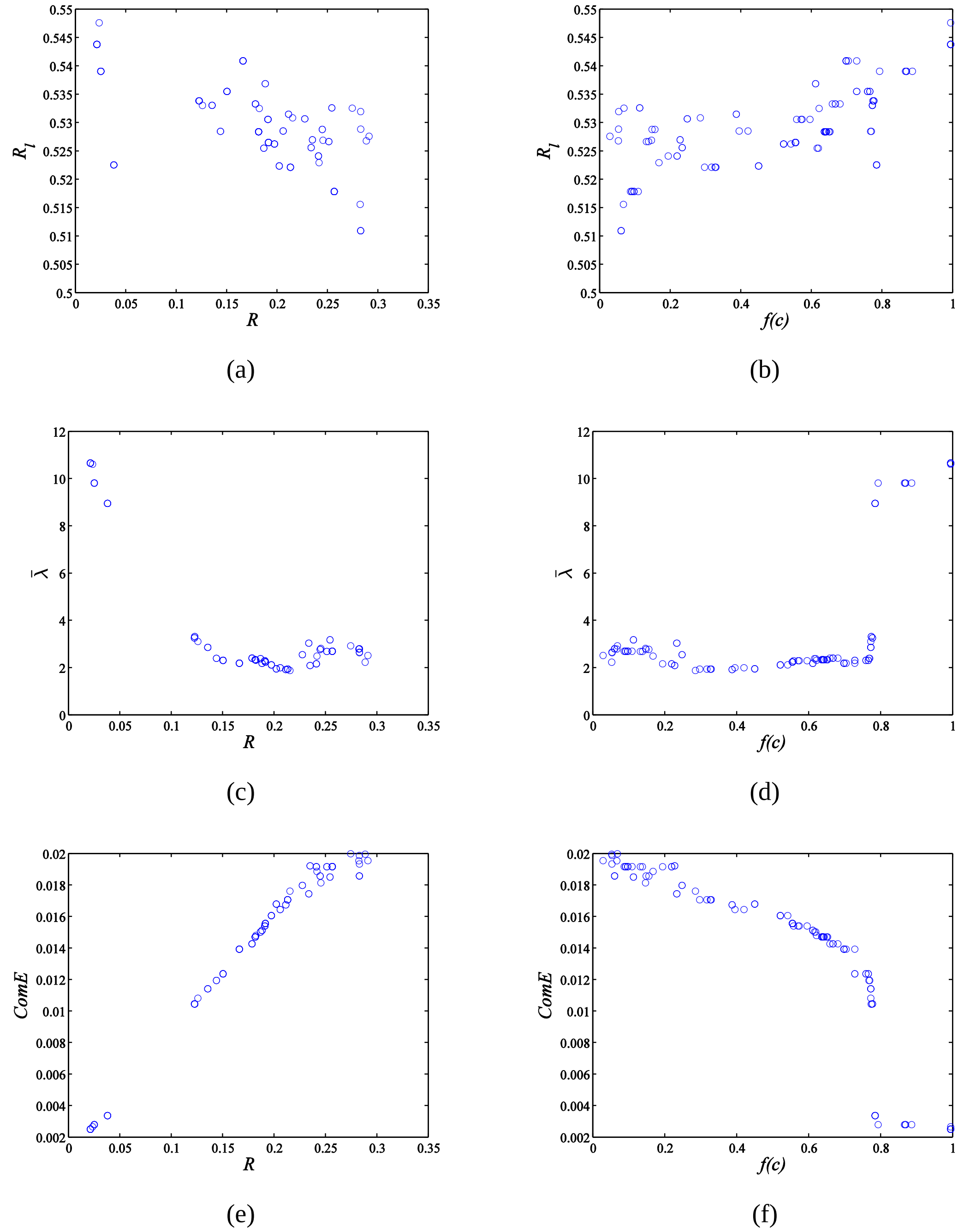

Supplementary Figure S9. Numerical results of the robustness of networks on Pareto fronts in Fig. 2: (a) and (b) are evaluated by R_l, (c) and (d) are evaluated by $\bar{\lambda}$, and (e) and (f) are evaluated by *ComE* (the definitions of these measures are given in Supplementary Note 1).

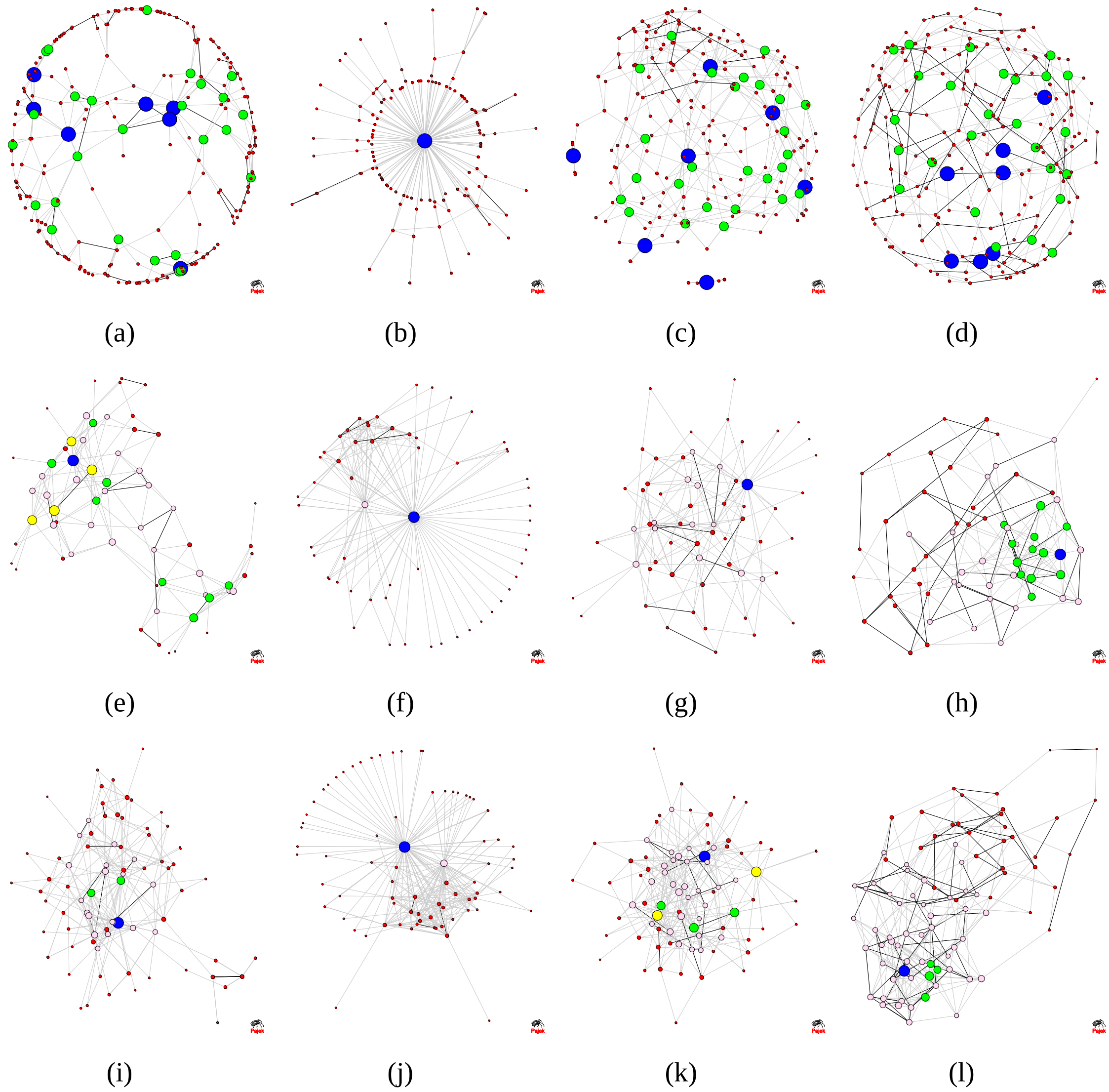

Supplementary Figure S10. Original structures of the three real world networks and topologies of extracted networks from the Pareto fronts in Fig. 5. Images in the first column represent the original structures of real world networks: (a) is WU Power grid network, (e) is Dolphin social network, and (i) is Scotland corporate interlock network. Other images in the first row ((b), (c), and (d)) are extracted from Pareto front initialized by network in (a), in the second row ((f), (g), and (h)) are initialized by network in (d), and in the last row ((j), (k), and (l)) are initialized by network in (i), and they represent for G_l, G_m, and G_r separately. Also, in the figures, the size of nodes is proportional to the degree of this node, and links between nodes with same degree are highlighted.

Supplementary Table

The detail of the real world networks studied in this paper are presented in Supplementary Table S1, which includes the number N of nodes, the number M of links, and description of the networks.

Supplementary Table S1. Detail information of the real world networks analyzed in the paper.

Name	N	M	Description
WU Power grid network[S4]	217	320	West European power network.
Dolphin social network [S5]	62	159	Social network of bottlenose dolphins living in New Zealand.
Scotland corporate interlock network[S6]	86	273	Corporate interlock network in Scotland during 1904-1905

The parameters used in MOEA-Net$_{rc}$ are described in Supplementary Table S2.

Supplementary Table S2. Parameter settings of MOEA-Net$_{rc}$.
("-" means determined by the algorithm in the running process)

Parameter	Meaning	Value
Ω	The size of population	200
$Maxgen$	The maximum generation	300
p_m	Mutation rate	0.1
P	Initialized population	-
F	Non-dominated individuals	-
Q	Temporary population	-
EP	External population restoring non-dominated solutions	-

Supplementary References

[S1] Zeng, A. & Liu, W. Enhancing network robustness against malicious attacks. *Physical Review E* **85**, 066130 (2012).

[S2] Wu, J., Barahona, M., Tan, Y. J. & Deng, H. Z. Spectral measure of structural robustness in complex networks. *IEEE Trans. Syst. Man Cybern. A: Syst. and Humans* **41**, 1244-1252 (2011).

[S3] Latora, V. & Marchiori, M. Efficient behavior of small-world networks. *Physical Review L* **87**, 198701 (2001).

[S4] Zhou, Q. & Bialek, J. W. Approximate model of European interconnected system as a benchmark system to study effects of cross-border trades. *IEEE Trans. on Power Sy stems* **20**, 782-788 (2005).

[S5] Lusseau, D. *et al.* The bottlenose dolphin community of doubtful sound features a large proportion of long-lasting associations. *Behav. Ecol. Sociobiol.* **54**, 396-405 (2003).

[S6] Scott, J. & Hughes, M. *The Anatomy of Scottish Capital: Scottish Companies and Scottish Capital, 1900–1979* (*Croom Helm*, London, 1980).
